# Genome-Wide CRISPR Screen Identifies Host Factors Required by *Toxoplasma gondii* Infection

**DOI:** 10.3389/fcimb.2019.00460

**Published:** 2020-01-22

**Authors:** Shui-Zhen Wu, Hai-Xia Wei, Dan Jiang, Sheng-Min Li, Wei-Hao Zou, Hong-Juan Peng

**Affiliations:** Guangdong Provincial Key Laboratory of Tropical Disease Research, Department of Pathogen Biology, School of Public Health, Southern Medical University, Guangzhou, China

**Keywords:** *Toxoplasma gondii*, host dependency factors, CRISPR-Cas9, genome-wide, actin cytoskeleton, immune regulation

## Abstract

*Toxoplasma gondii* are obligate intracellular protoza, and due to their small genome and limited encoded proteins, they have to exploit host factors for entry, replication, and dissemination. Such host factors can be defined as host dependency factors (HDFs). Though HDFs are inessential for cell viability, they are critical for pathogen infection, and potential ideal targets for therapeutic intervention. However, information about these HDFs required by *T. gondii* infection is highly deficient. In this study, the genes of human foreskin fibroblast (HFF) cells were comprehensively edited using the lentiviral CRISPR-Cas9-sgRNA library, and then the lentivirus-treated cells were infected with *T. gondii* at multiplication of infection 1 (MOI = 1) for 10 days to identify HDFs essential for *T. gondii* infection. The survival cells were harvested and sent for sgRNA sequencing. The sgRNA sequence matched genes or miRNAs were potential HDFs. Some cells in the lentivirus-treated group could survive longer than those in the untreated control group after *T. gondii* infection. From a pool of 19,050 human genes and 1,864 human pri-miRNAs, 1,193 potential HDFs were identified, including 1,183 genes and 10 pri-miRNAs (corresponding with 17 mature miRNAs). Among them, seven genes and five mature miRNAs were validated with siRNAs, miRNA inhibitors, and mimics, respectively. Bioinformatics analysis revealed that, among the 1,183 genes, 53 potential HDFs were associated with regulation of host actin cytoskeleton and 23 potential HDFs coded immune negative regulators. This result indicated that actin dynamics were indispensable for *T. gondii* infection, and some host immune negative regulators may be involved in disarming host defenses. Our findings contribute to the current limited knowledge about host factors required by *T. gondii* infection and provide us with new targets for medication therapy and vaccine exploitation.

## Introduction

*Toxoplasma gondii* is an obligate intracellular protozoa that is considered one of the most intelligent parasites with numerous and widespread hosts (Kieffer and Wallon, 2013). The infection in an immune competent individual is usually asymptomatic but results in encephalitis, retinochoroiditis, and other serious diseases in immunocompromised individuals (Kieffer and Wallon, 2013). When pregnant women are primarily infected by *T. gondii*, abortion, fetal anomalies, stillbirth, and other adverse outcomes may occur (Li et al., 2014). So far, there is no ideal therapeutic medicine or vaccine against *T. gondii* infection.

The pathology of toxoplasmosis is a direct or indirect result of the parasite's lytic cycle of attachment, invasion, parasitophorous vacuole (PV) formation, growth, and egress (Black and Boothroyd, 2000; Peng et al., 2011). *Toxoplasma gondii* are obligate intracellular protozoa, and due to their small genomes and a limited number of encoded proteins, they have to exploit host factors for entry, replication, and dissemination. Such host factors can be defined as host dependency factors (HDFs). Though knowledge about the HDFs supporting *T. gondii* infection is highly deficient, some amino acids, host proteins, and miRNAs have been identified to be utilized by *T. gondii* during infection. For example, as *T. gondii* lacks the enzymes for polyamine, arginine, and purine biosynthesis, the intracellular parasites have to get these nutrients from their host cells (Schwartzman and Pfefferkorn, 1982; Fox et al., 2004; Cook et al., 2007). We previously reported that host RhoA and Rac1 GTPases were activated upon *T. gondii* infection and facilitated *T. gondii* invasion (Na et al., 2013; Wei et al., 2019). It also has been reported that host hypoxia-inducible transcription factor 1 (HIF-1)/hexokinase 2 (HK2) can reprogram the host cell's metabolism to create an environment conducive for parasite replication at 3% physiological oxygen levels (Menendez et al., 2015). Epidermal growth factor receptor (EGFR) is reported to be activated by *T. gondii* to maintain the non-fusogenic nature of the PV and prevent the autophagy pathway–dependent killing of the parasite (Muniz-Feliciano et al., 2013).

Previously, three papers reported that genome-wide RNAi screening was performed to identify host genes required for *T. gondii* growth (Gaji et al., 2013; Moser et al., 2013; Menendez et al., 2015). However, the overlap of these genes was very poor. Six human proteins (PTK9L, MAPK7, PHPT1, MYLIP, PTPRR, and PPIL2) have been reported to facilitate *T. gondii* entry by modifying host actin dynamics (Gaji et al., 2013). It has been suggested that the screened potential HDFs for *T. gondii* are also vital for the host cells (Moser et al., 2013). Menendez et al. identified 316 and 293 siRNAs that could reduce the parasite's growth under 21 and 3% O_2_ conditions, respectively, and HK2 functions as a very important HDF for *T. gondii* infection at physiological oxygen levels (Menendez et al., 2015).

The CRISPR-cas9 system has recently been widely used as a tool to screen HDFs for virus infection, including human immunodeficiency virus (HIV), West Nile virus (WNV), dengue virus (DENV), Zika virus (ZIKV) (Ma et al., 2015; Zhang et al., 2016; Park et al., 2017), and tumor growth (Shi et al., 2015; Steinhart et al., 2017; Yau et al., 2017). Compared with RNAi technology, the gene-silencing efficiency of the CRISPR system is much higher, and the target genes can be completely deleted. In our study, the whole-genome lentiviral CRISPR-Cas9-sgRNA library was used to identify HDFs essential for *T. gondii* infection. This study will contribute to the current limited knowledge about host factors required by *T. gondii* infection and provide us with new targets for therapy and vaccine exploitation.

## Materials and Methods

### *Toxoplasma gondii* Strains and Cell Lines

The *T. gondii* wild-type ME49 strain tachyzoites were maintained in our laboratory. The human foreskin fibroblast (HFF) and Hela cell lines were purchased from the American Type Culture Collection (ATCC) (USA). Parasites and cells were cultured in Dulbecco's modified Eagle's medium (DMEM) (Gibco, New York, NY) containing 10% (v/v) fetal bovine serum (FBS) (Gibco, New York, NY) in 5% CO_2_ at 37°C.

### Lentiviral Transduction, *T. gondii* Infection, and sgRNA Sequencing

**Figure 1:**
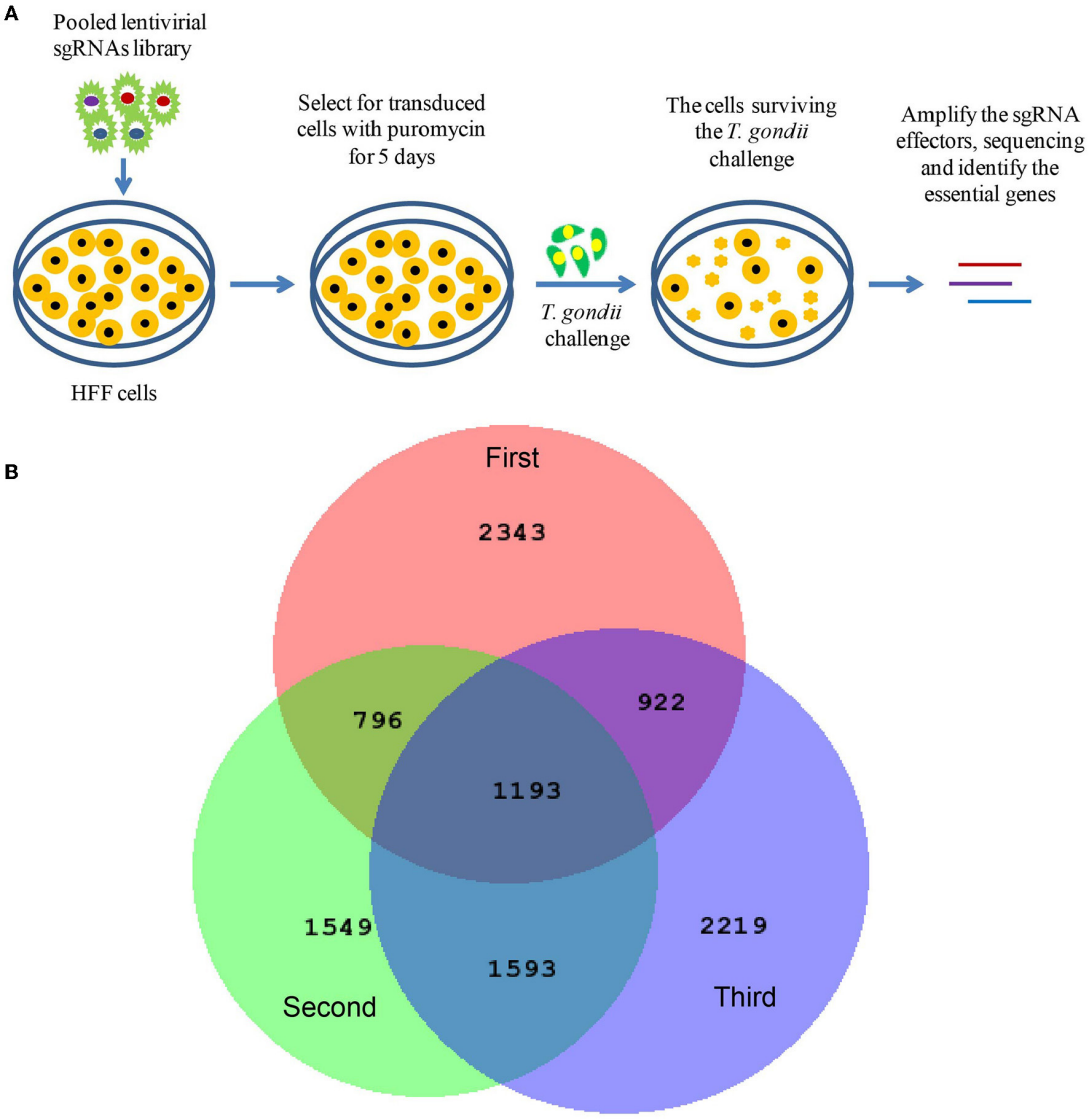
Screening of host dependency factors required by *Toxoplasma gondii* infection with CRISPR/CAS9-sgRNA library. **(A)** Screening flowchart. Human foreskin fibroblast (HFF) cells were transduced with lentiviruses at multiplicity of infection 1 (MOI = 1). Cells were subsequently selected using puromycin for 5 days. Transduced cells were infected with *T. gondii* (MOI = 1) for 10 days. Finally, the transduced cells that survived *T. gondii* infection were harvested, and the genomic DNA was extracted and subjected to high-throughput sequencing. **(B)** Venn diagram depicting the number of screened targets from independent biological triplicates: first replicate (red), second replicate (lime), third replicate (blue), and overlapped targets (middle).

A lentiviral CRISPR-Cas9-sgRNA library targeting 19,050 human genes with 6 sgRNAs per gene and 1,864 pri-miRNAs with 4 sgRNAs per miRNA was derived from Zhang's lab (Sanjana et al., 2014; Shalem et al., 2014) and purchased from Shanghai GeneChem Company. The screening strategy is illustrated in Figure 1A, and the detail procedure was as follows. (1) HFF cells (6 × 10^7^) were transduced with lentiviruses at multiplicity of infection 1 (MOI = 1) for 72 h. (2) The cells were subsequently screened with puromycin for 5 days. (3) About 1 × 10^7^ HFF cells treated or untreated with the lentivirus library were infected with *T. gondii* ME49 tachyzoites (MOI = 1) and incubated for 10 days. *T. gondii* infection led to a small population of host cells carrying lentivirus-sgRNA being protected and surviving, and lysis of those normal HFF cells. (4) Genomic DNA was extracted from the cells that survived *T. gondii* infection, and sgRNA sequences were amplified by nested PCR with the outer primers first (forward: 5′-AATGGACTATCATATGCTTACCGTAACTTGAAAGTATTTCG-3′; reverse: 5′-AACGTTCACGGCGACTACTGCACTTATATACGGTTCTC-3′), and then the inner primers (forward: 5′-TCTTGTGGAAAGGACGAAACACCG-3′; reverse: 5′-AGCCAGTACACGACATCACTTTCC-3′). (5) After high-throughput sequencing, the tag sequences were removed and the sgRNAs were matched to their specefic genes. These genes are potential HDFs which may be essential for *T. gondii* infection. The experiment was done in biological triplicates.

### Screening of the Potential HDFs and Validation of the Selected HDFs

The sgRNAs were arranged by their reads and the top 39,000 sgRNAs cut off at the last one with the same reads were selected from the three independent biological replicates. These screened sgRNAs were further matched to their origin genes, only the candidate genes or pri-miRNAs with three or more matched independent sgRNAs in all three independent biological replicates were selected. Then, seven genes were further verified by siRNA gene knockdown assay, five mature miRNAs were validated with specific inhibitors and mimics. All siRNAs were purchased from Ribobio Company (Guangzhou, China) and used at 100 nM working concentration. All inhibitors and mimics were purchased from GenePharma Company (Shanghai, China) and used at 10 nM working concentration. Hela cells were transfected with siRNAs, inhibitors, and mimics against the candidate genes using Lipofectamine 2000 (Invitrogen, cat. #11668-019) for 24 h. In parallel, the control siRNA, inhibitor, and mimic were also transfected to Hela cells for control. Then the transfected cells were infected with *T. gondii* (MOI = 1) for 36 h. The infected cells were collected and treated with Proteinase K (Qiagen, cat. #19131). To evaluate the relative amount of *T. gondii* tachyzoites, *T. gondii* B1 gene was detected by real-time PCR using the specific primers (forward: 5′-GGAACTGCATCCGTTCATG-3′; reverse: 5′-TCTTTAAAGCGTTCGTGGTC-3′). The standard curve was obtained through real-time PCR with B1 gene primers and the plasmid containing B1 gene of known concentrations as the template. Abundance of the parasitic equivalent was determined by extrapolation from the standard curve. The experiments were prepared in triplicates and performed for three times. The information for gene-specific and control siRNAs, inhibitors, and mimics is provided in Table S1.

### Cell Viability Assay

Cell viability was assessed by a cell counting kit (CCK; Transgen, cat. #FC101) following the manufacturer's instructions. Equal numbers of Hela cells were seeded in the wells of 96-well plates. When the cells reached 80% confluency, they were transfected with siRNAs, inhibitors, or mimics and incubated for 24 h. Then, 10 μl CCK solution was added to each well and the cells were incubated for 4 h at 5% CO_2_, 37°C. The absorbance was measured at 450 nm using a microplate reader (BioTek). The experiments were prepared in triplicates and performed for three times.

### Gene Ontology Enrichment and KEGG Pathway Analysis

To further define the biological function of the identified potential HDF genes, Gene Ontology (GO) and Kyoto Encyclopedia of Genes and Genomes (KEGG) pathway enrichment were done using the online server Enrichr (http://amp.pharm.mssm.edu/Enrichr) (Kuleshov et al., 2016). Additionally, the STRING database (version 11.0; http://string-db.org) was used to explore and build the protein–protein interaction (PPI) network. Targetscan (Agarwal et al., 2015), miRWalk (Dweep et al., 2014; Sticht et al., 2018), and DIANA-microT (Paraskevopoulou et al., 2013) were used to predict the target genes of the mature miRNAs.

### Statistical Analysis

The differences between two groups were analyzed with Prism (GraphPad Software) using a 2-tailed Student's *t*-test with unequal variance. To compare more than two groups, one-way ANOVA and least significant difference (LSD) test were performed with the SPSS software package. The statistical significance was defined as *p* < 0.05.

## Results

### Genome-Wide CRISPR Screen for *T. gondii* Dependency Factors

HFF cells were transduced with lentiviruses and subsequently selected using puromycin. Then, the cells were infected with *T. gondii* (MOI = 1). At 4 days post infection, more free tachyzoites were found in the lentivirus-treated group. This result may be due to the silencing of some immune-associated genes. At 10 days post-infection, the cells in the normal group were all lysed; however, there were still remaining cells in the lentivirus-treated group (Figure S1). The genomic DNA was extracted from the cells that survived *T. gondii* infection and subjected to high-throughput sequencing. Similar to RNAi screening, we used the reagent redundancy principle (Echeverri et al., 2006) to select the candidate genes or pri-miRNAs for each independent screen. The sgRNAs were arranged by reads, and the top 39,000 sgRNAs cut off at the last one with the same reads were selected, thus 36,898, 36,901, and 39,133 sgRNAs were chosen from the three independent biological replicates, respectively (Table S2). Only those genes or pri-miRNAs targeted by three or more independent sgRNAs in each independent biological replicate were further screened. Therefore, 5,149, 4,988, and 5,763 genes and 105, 143, and 164 pri-miRNAs were selected from the three independent biological replicates (Table S3). In total, 1,183 genes and 10 pri-miRNAs were found to be overlapped in all the three independent biological replicates, and the 10 pri-miRNAs were found with 17 mature miRNAs (Figure 1B and Table S3). Among the 1,183 genes, EGFR, which had been previously confirmed as an HDF required by *T. gondii* to prevent autophagy protein-mediated killing of the parasites (Muniz-Feliciano et al., 2013), was also identified in our study. In addition to these known factors, the majority of the genes were newly defined potential HDFs for *T. gondii* in our study. Notably, to our knowledge, this is the first report of host dependency miRNAs required by *T. gondii*. In all, 2,760 genes were predicted to be targeted by the 17 mature miRNAs (Table S4).

### Validation of *T. gondii* Dependency Host Factors

**Figure 2:**
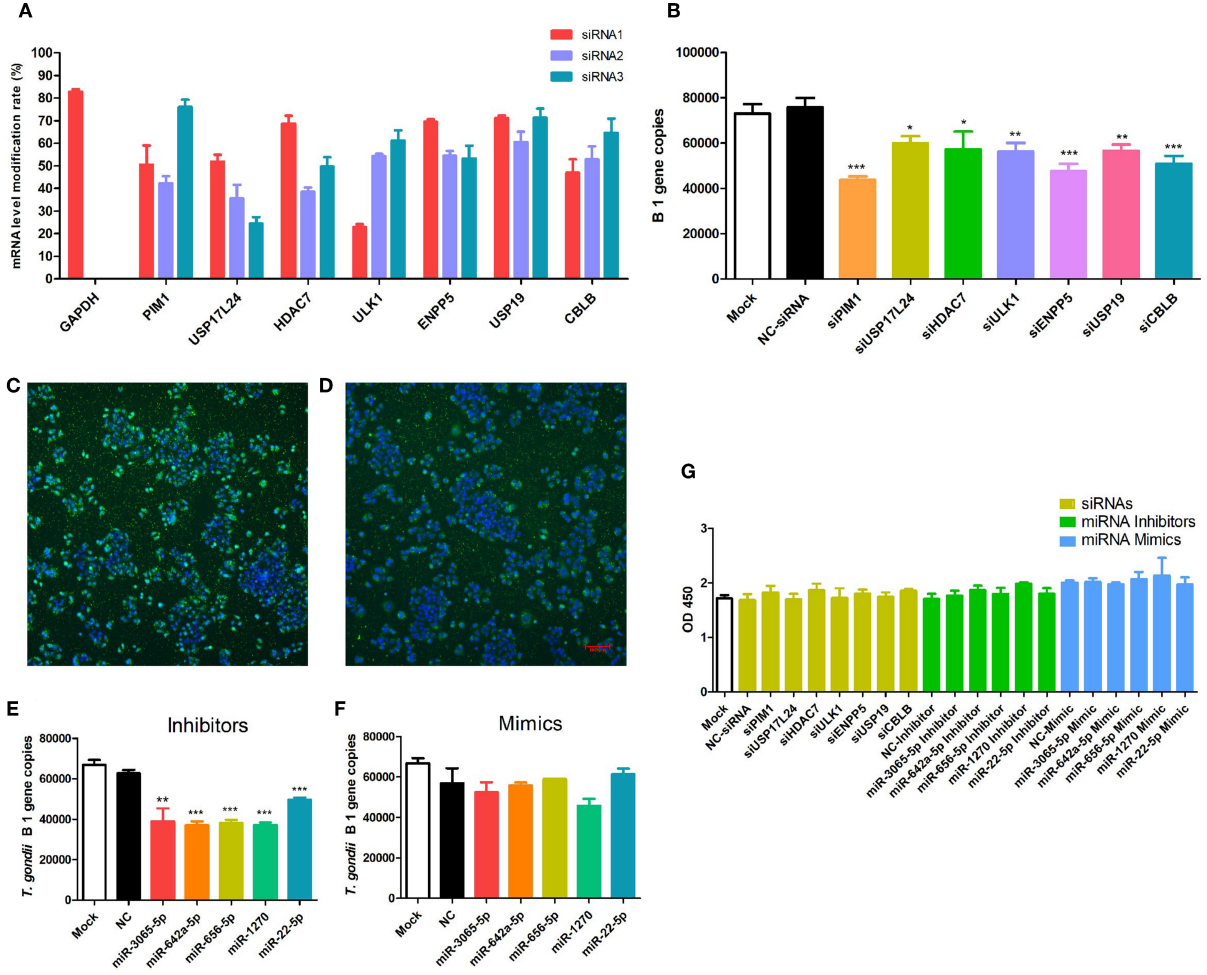
Validation of seven selected *T. gondii* host dependency factors (HDFs). **(A)** Among the HDFs, seven genes (CBLB, USP17L24, USP19, HDAC7, ULK1, PIM1, and ENPP5) were selected for siRNA gene knockdown assay on Hela cells. Three siRNAs were designed for each gene, and siRNAs with the highest knockdown efficiency were chosen for the further experiments. **(B)** When specific genes were knocked down with selected siRNA for 24 h, *T. gondii* was used to infect cells at MOI = 1 for 36 h. Proliferation of *T. gondii* was significantly inhibited in siRNA treated groups compared with mock and negative control siRNA treated groups. **(C,D)** FAM-tagged control miRNA inhibitor and mimic transfected Hela cells showed the transfection rate. **(E,F)** Compared with mock and negative control siRNA treated groups, multiplication of *T. gondii* was significantly suppressed in specific miRNA inhibitor treated group, but not significantly changed in mimic treated group. **(G)** Viability of Hela cells in siRNA, inhibitor, and mimic treated groups was not significantly changed. * *p* < 0.05, ** *p* < 0.01, *** *p* < 0.005.

To further validate the factors identified by genome-wide CRISPR screen, seven genes (CBLB, USP17L24, USP19, HDAC7, ULK1, PIM1, and ENPP5) were selected for siRNA gene knockdown assay. Three siRNAs were designed for each gene, and the knockdown efficiency is shown in Figure 2A. Then the siRNAs with the highest knockdown efficiency were chosen for each gene in the next experiments. After the specific genes were knocked down by the specific siRNAs, *T. gondii* ME49 strain was used to infect the cells. As shown in Figure 2B, the proliferation of *T. gondii* was significantly inhibited in the siRNA treated groups. Similarly, five mature miRNAs (miR-1270, miR-22-5p, miR-3065-5p, miR-642a-5p, and miR-656-5p) were validated with their specific inhibitors and mimics. The transfection rates were indicated with the FAM-tagged control inhibitors and mimics (Figures 2C,D). When Hela cells were treated with specific miRNA inhibitors, the multiplication of *T. gondii* was significantly suppressed (Figure 2E). However, when Hela cells were treated with specific miRNA mimics, the proliferation of *T. gondii* was not significantly affected (Figure 2F). The viability of Hela cells was not significantly changed in the siRNA, inhibitor, and mimic treated groups (Figure 2G).

### Bioinformatics Analysis of the Screened *T. gondii* HDFs

**Figure 3:**
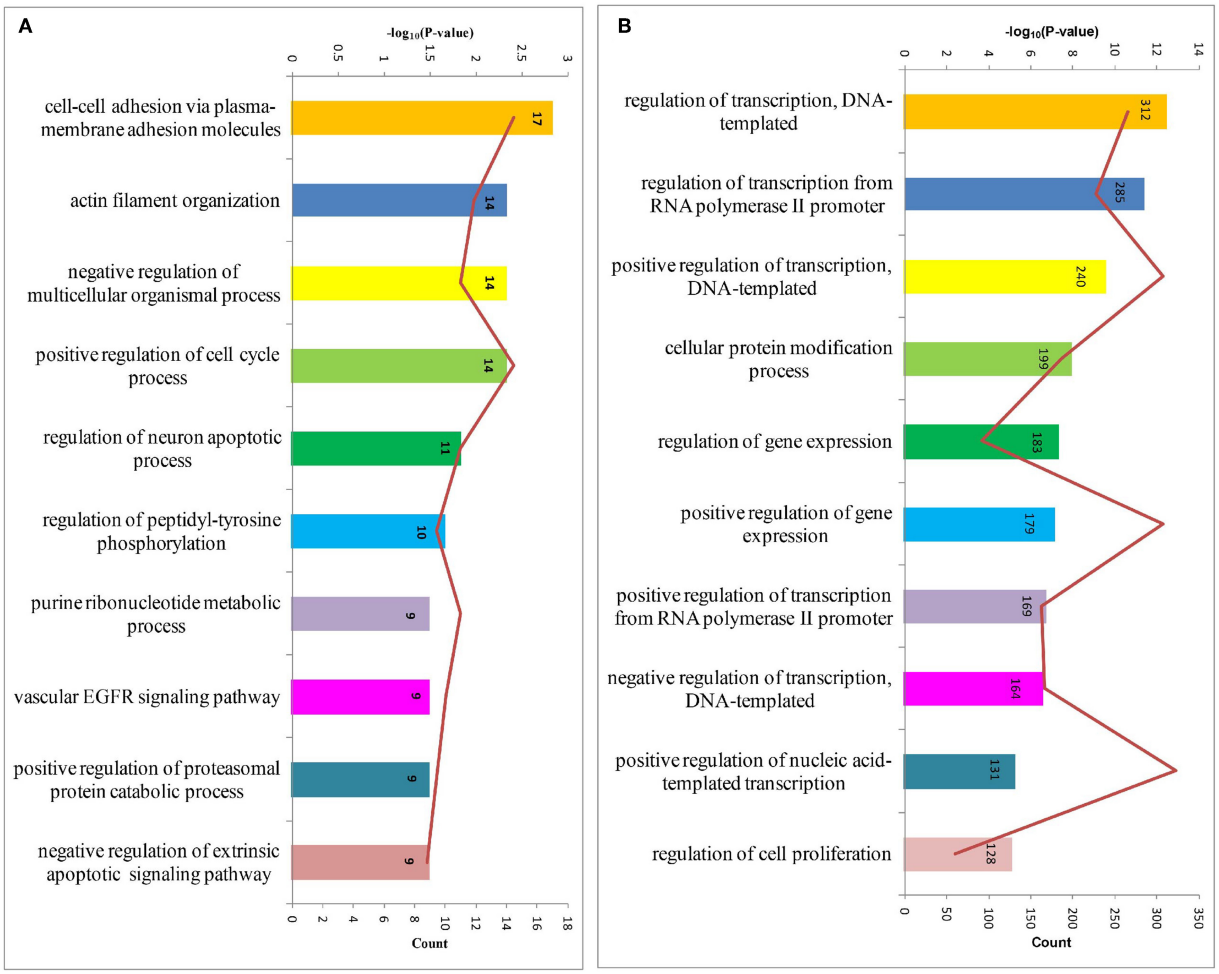
The top 10 enriched biological processes for host dependency genes and predicted target genes of host dependency miRNAs identified by Gene Ontology (GO) analysis. **(A)** Host dependency genes were significantly over-represented in the biological processes of actin filament organization, negative regulation processes, and regulation of apoptotic processes. **(B)** Host dependency miRNA predicted target genes were involved in a variety of positive regulation process, such as “positive regulation of transcription, DNA-templated” and “positive regulation of gene expression.” Stacked bar chart indicates the number of proteins overlapped with the database, and connected orange points represent the logarithm of *p*-values.

In order to obtain a comprehensive view of the screened HDFs, we performed GO analysis to identify the significantly enriched functional terms. The results revealed that the 1,183 screened HDFs were significantly enriched in a variety of biological processes (*p* < 0.05). The top 10 enriched terms within the Biological Process category were as follows, cell-cell adhesion via plasma-membrane adhesion molecules, actin filament organization, negative regulation of multicellular organismal process, positive regulation of cell cycle process, regulation of neuron apoptotic process, regulation of peptidyl-tyrosine phosphorylation, purine ribonucleotide metabolic process, vascular EGFR signaling pathway, positive regulation of proteasomal protein catabolic process, and negative regulation of extrinsic apoptotic signaling pathway (Figure 3A). The top 10 enriched terms within the Biological Process category for the predicted target genes of the 2,760 host dependency miRNAs were involved in a variety of positive regulation process, such as positive regulation of transcription, DNA-templated, positive regulation of gene expression, positive regulation of transcription from RNA polymerase II promoter, positive regulation of nucleic acid-templated transcription, and so on (Figure 3B). These results show that potential host dependency genes may be important for negative regulation, while host dependency miRNA predicted targets are important for positive regulation.

**Figure 4:**
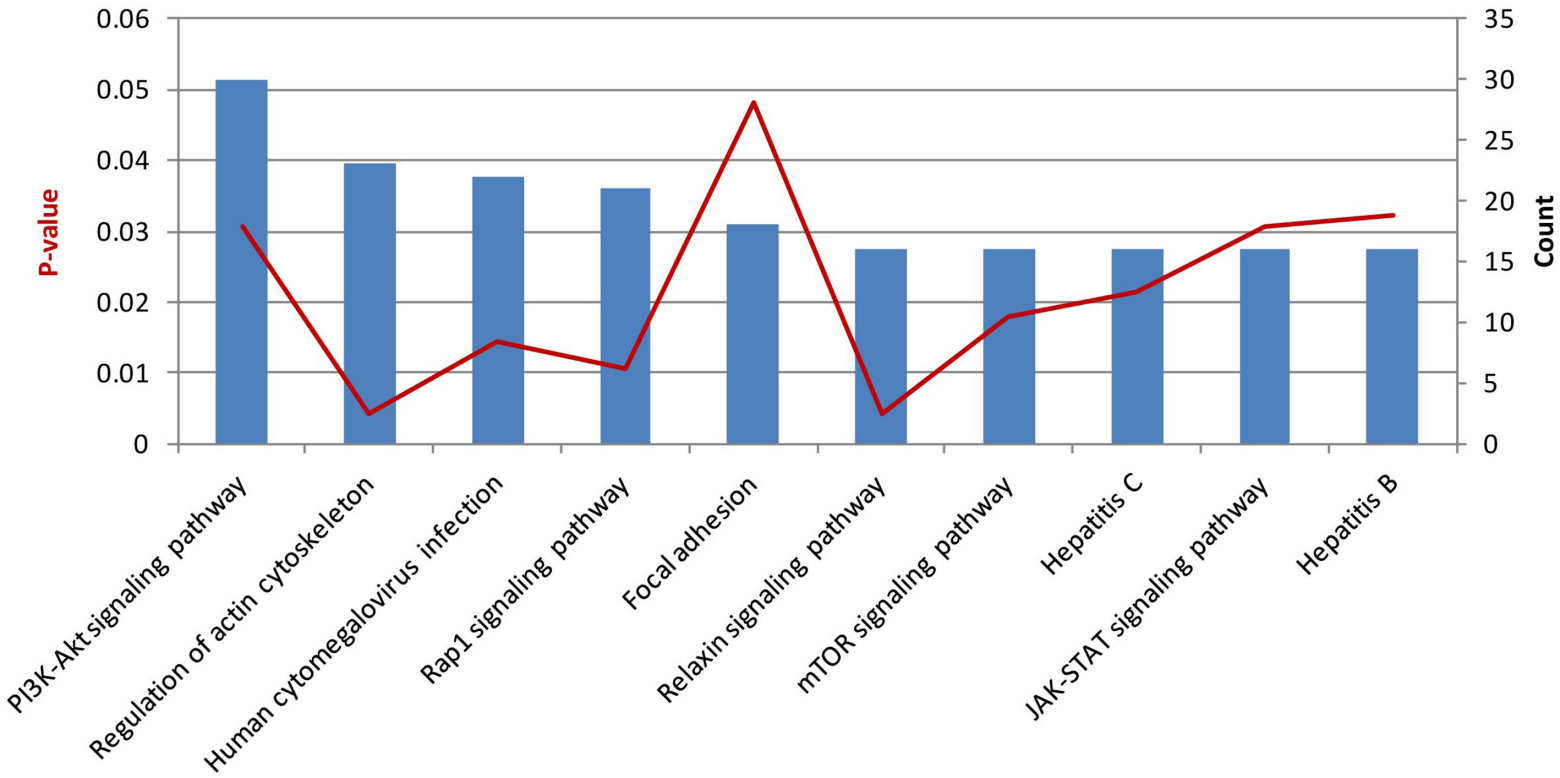
The top 10 enriched pathways for host dependency genes. Stacked bar chart indicates the number of proteins overlapped with the database, and connected orange points represent *p*-values.

**Figure 5:**
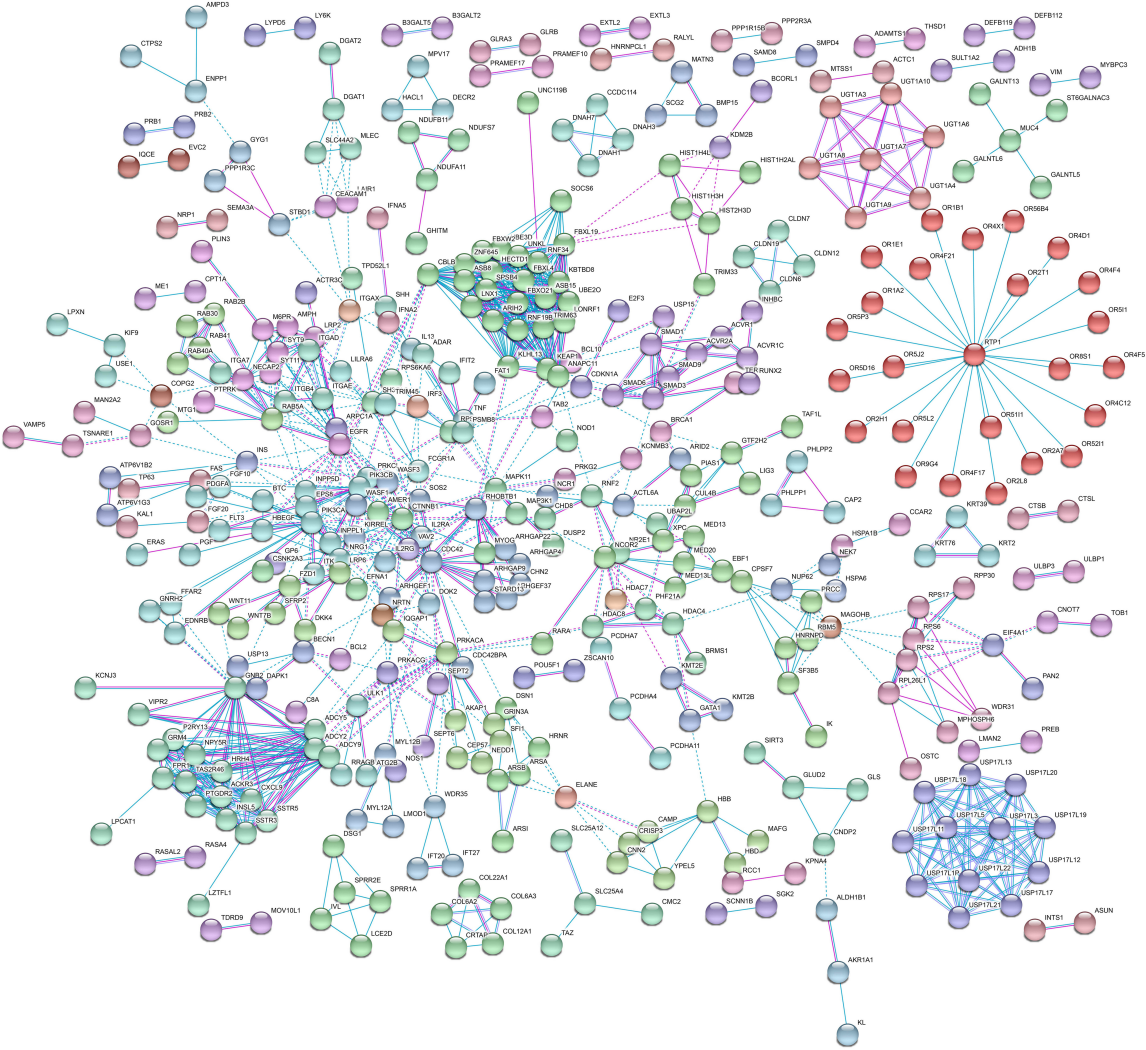
Protein–protein interaction (PPI) network of host dependency genes. Among the 1,183 proteins, 428 (36.12%) of host dependency genes were enriched in a large PPI network, with 1,136 edges. Seven PPI clusters were evident in the network. The biggest one is a cluster of E3 ubiquitin-protein ligases containing 25 proteins (in green), including TRIM63, UBE3D, HECTD1, ARIH2, ZNF645, and CBLB, was observed as one of the main hubs. The second one is a cluster of 25 olfactory receptor proteins (in red) with RTP1 in the center. A cluster of 12 USP proteins (in medium slate blue) was also found protruding. Detailed information on these seven PPI clusters is shown in Table S6.

Then, we performed KEGG pathway enrichment to further investigate the significant human signaling pathways required by *T. gondii* infection. The top 10 enriched pathways for the 1,183 identified potential host dependency genes are shown in Figure 4 and Table S5. We found 30 genes enriched in the PI3K-Akt signaling pathway, 21 genes in the Rap1 signaling pathway, 16 genes in the mTOR signaling pathway, 16 genes in the JAK-STAT signaling pathway, 23 genes in regulation of actin cytoskeleton, 16 genes in the relaxin signaling pathway, 18 genes in focal adhesion, 22 genes in human cytomegalovirus infection, 16 genes in hepatitis C, and 16 genes in hepatitis B. These results indicate that the identified HDFs are important for infectious diseases and cytoskeleton reorganization. We further conducted a deeper exploration of the PPI networks by using the STRING 11.0 database to elucidate whether these potential HDFs were functionally related. Those reported experimental data and prediction databases were used as active interaction sources. By applying a high confidence of *p* > 0.7, 428 (36.12%) of the host dependency genes were enriched in a large PPI network, with a PPI enrichment *p* of 4.82e^−07^ (Figure 5), indicating that the screened HDFs were biologically connected as a network rather than a random set of proteins. As shown in Figure 5, 1,136 edges (PPIs) were observed among the 428 HDFs, and seven protein–protein-interacting clusters were evident in the network (Table S6). Among these, the biggest one was a cluster of E3 ubiquitin-protein ligases containing 25 proteins (in green), including TRIM63, UBE3D, HECTD1, ARIH2, ZNF645, and Casitas B-lineage lymphoma-b (CBLB) etc, notably, CBLB was observed as one of the main hubs situated in the core of this cluster. Then the second one was a cluster of 25 olfactory receptor proteins (in red) with receptor-transporting protein 1 (RTP1) in the center. A cluster of 12 ubiquitin-specific proteases (USP) (in medium slate blue) was also found among the seven PPI clusters.

## Discussion

Obligate intracellular parasites rely on host resources for entry, replication, and dissemination. Previously, three papers reported on identification of host genes required for *T. gondii* growth through genome-wide RNAi screening, revealed that host HK2 was essential for *T. gondii* growth under 3% physiological oxygen concentration, 6 human proteins (PTK9L, MAPK7, PHPT1, MYLIP, PTPRR, and PPIL2) facilitated *T. gondii* entry by modifying host actin dynamics (Gaji et al., 2013; Moser et al., 2013; Menendez et al., 2015). In our study, the human CRISPR-cas9 sgRNA library was used to screen HDFs for *T. gondii* infection. Finally, 1,183 genes and 10 pri-miRNAs were identified as HDFs. Among them, seven genes (CBLB, USP17L24, USP19, HDAC7, ULK1, PIM1, ENPP5) and five mature miRNAs (miR-1270, miR-22-5p, miR-3065-5p, miR-642a-5p, miR-656-5p) were further confirmed by siRNA knockdown assay or miRNA inhibitors. Furthermore, EGFR, which had been previously confirmed as an HDF required by *T. gondii* preventing autophagy protein and lysosomal protease mediated killing of the parasite (Muniz-Feliciano et al., 2013), was identified in our study. All of these results strongly support the reliability of our CRISPR-cas9 system. The majority of the 1,183 genes and 10 human pri-miRNAs were newly screened HDFs required by *T. gondii* infection.

### The Screened HDFs Function in Actin Reorganization

It is known that cytoskeletal reorganization is induced when cells are invaded by bacteria, viruses, or protozoan parasites. For example, the bacteria *Salmonella enterica* induces host cell actin reorganization to facilitate its internalization in non-phagocytic cells (Patel and Galan, 2005). The vaccinia virus utilizes polymerized actin to invade non-infected cells (Munter et al., 2006). The protozoan *Cryptosporidium parvum* induces host actin reorganization to form a plaque-like structure at the host–parasite interface during parasite invasion and persists during parasite replication (Elliott and Clark, 2000). *Toxoplasma gondii* infection induces *de novo* polymerization of the host cell actin, and F-actin is observed to accumulate and form a ring-shaped structure at the point of apposition of the host and parasite plasma membranes, which remains stable during the parasite's entry and disappears from the posterior end of the internalized parasite within 10 min post entry (Gonzalez et al., 2009). After that, *T. gondii* recruits host cell microtubules to form conduits, along which host organelles are transported to the PV (Coppens et al., 2006). The RacGTPases were activated upon *T. gondii* invasion, and when F-actin polymerization was inhibited in NSC23766-treated cells, the efficiency of *T. gondii* attachment, invasion, and replication was significantly decreased (Na et al., 2013; Wei et al., 2019). Furthermore, it has been reported that six human proteins (PTK9L, MAPK7, PHPT1, MYLIP, PTPRR, and PPIL2) facilitate *T. gondii* entry by modifying host actin dynamics (Gaji et al., 2013). In our study, among the HDF genes we screened, 14 genes (MYBPC3, INPPL1, LMOD1, MYBPH, PPP1R9A, ERMN, RHOBTB1, EPS8, CDC42, SAMD14, ACTC1, SH3BP1, FAT1, CAP2) were significantly enriched in “actin filament organization” in the GO term of Biological Process (*p* = 0.012). In KEGG pathway enrichment, 23 genes fell in “regulation of actin cytoskeleton” (*p* = 0.0041), and 16 genes fell in “relaxin signaling pathway” (*p* = 0.0043). All these results further testify that cytoskeletal reorganization is essential for *T. gondii* infection.

### Screened HDFs May Negatively Regulate Host Immunity

During pathogen infection, immune defense is strongly induced in the host cells. Both innate and adaptive immune defense play essential roles in pathogen elimination. However, there exists a variety of negative regulation signaling to avoid excessive pro-inflammatory reaction and pathological damage (Liu et al., 2005; O'Neill, 2008). Moreover, immune tolerance is a normal phenomenon in many chronic infections (Han et al., 2013; Larrubia et al., 2013; Snell et al., 2017; Dembek et al., 2018; Teymouri et al., 2018). Therefore, this kind of negative regulation of host cell immunity can be employed by intracellular pathogens for their immune evasion.

In this study, two of the top 10 enriched GO terms within Biological Process for the screened host dependency genes were about negative regulation processes negative regulation of multicellular organismal process, and negative regulation of extrinsic apoptotic signaling pathway (Figure 3A). On the other hand, among the predicted target genes of the host dependency miRNAs, four of the top 10 enriched GO terms within Biological Process were involved in various positive regulation processes including positive regulation of transcription, DNA-templated, positive regulation of gene expression, positive regulation of transcription from RNA polymerase II promoter, and positive regulation of nucleic acid-templated transcription (Figure 3B). These results indicate that the screened host dependency miRNAs targeted the genes and suppressed their functions on positive regulation of host immunity. Surprisingly, 23 of the screened 1,183 host dependency genes were reported to contribute to immune tolerance. As shown in Table 1, the majority of the genes facilitate immune tolerance by inhibiting T or B cell function, and some of them can negatively regulate innate immunity, such as ADAR and CBLB. Interestingly, PIM kinases are reported to promote viral infectivity by counteracting the host antiviral system; SIRT1/2 knockdown increased LPS and poly (I: C) induced IL-6, TNF-α, and MCP-1 levels. All these genes were screened as HDFs of *T. gondii* with our CRISPR-cas9 system, indicating that they may play essential roles in the negative regulation of host anti-*T. gondii* immunity.

**Table 1:** The 23 screened host dependency genes of *T. gondii* contributing to immune tolerance.

**Gene**	**Functions**	**References**
ADAR	T cell self-tolerance; a feedback suppressor of innate immunity	O'Connell et al., 2015; George et al., 2016; Chung and Rice, 2018
Bcl10	B cell tolerance	Yu et al., 2012
Bcl2	B-cell tolerance	Tsubata, 2017
C1qa	Facilitate the induction of intranasal tolerance	Baruah et al., 2010
CBLB	T- cell dysfunction, and negatively regulate innate immunity	Liu et al., 2014; Tang et al., 2019
CD84	B cell tolerance	Wong et al., 2015; Cuenca et al., 2019
CDC42	Suppress Th17 aberrant differentiation/pathogenicity and promote of Treg differentiation/stability/function	Kalim et al., 2018
CEACAM1	T-cell inhibition; deletion of CEACAM1 in mice causes exacerbation of inflammation and hyperactivation of myeloid cells and lymphocytes	Huang et al., 2015; Horst et al., 2018
DOK2	Involved in an intrinsic negative feedback loop downstream of NK-cell-activating receptors and early T cell receptor signal	Dong et al., 2006; Celis-Gutierrez et al., 2014
Fas	Maintenance of immune tolerance by regulating T cell and B cell immunity	Yamada et al., 2017
HDAC7	T cell self-tolerance	Verdin et al., 2004; Kasler et al., 2012
IL4I1	Inhibits T-cell proliferation and production of IFN-γ and inflammatory cytokines	Marquet et al., 2010; Scarlata et al., 2015
LRP6	Decreased effector T cell differentiation and increased regulatory T cell differentiation	Hong et al., 2016
NFIL3	T-cell dysfunction	Zhu et al., 2015; Kim et al., 2019
NRP1	A higher proportion of Nrp1^−/−^ Regulatory T cells produce IFNγ than (Nrp1^+/+^) Tregs.	Overacre-Delgoffe et al., 2017
PIM1	Facilitates promotion of viral infectivity by counteracting the host antiviral system	Miyakawa et al., 2019
PRKCI	In human ovarian cancers, high PRKCI expression correlates with high expression of TNFα and YAP1 and low infiltration of cytotoxic T cells	Sarkar et al., 2017
SEMA3A	Suppressing the over-activity of both T and B cell autoimmunity	Vadasz and Toubi, 2014; Cozacov et al., 2017
Sh2b3	Negatively regulates cytokine signaling in lymphohematopoiesis	Mori et al., 2018
SIRT2	Decreased LPS and poly (I:C) induced IL-6, TNF-α and MCP-1 expressions	Qin et al., 2017
SMAD3	Cytokine suppression	Yoshimura and Muto, 2011
SUMO4	Negatively regulates NF-kB transcriptional pathway	Park, 2007
TIGIT	Suppresses T cell activation; decreases IFN-γ production.	Yu et al., 2009; Lozano et al., 2012

In conclusion, our genome-wide CRISPR screen identified 1,193 HDFs required by *Toxoplasma gondii* infection, including 1,183 genes and 10 pri-miRNAs. The GO terms and KEGG enrichment for the host dependency genes and the target genes for the host dependency miRNAs revealed that these HDFs were characteristically involved in the regulation of host cell actin reorganization to facilitate *T. gondii* invasion, and in the modulation of negative host immunity for immune evasion. These findings provide us with new targets for therapy and vaccine exploitation.

## Data Availability Statement

The raw data supporting the conclusions of this article will be made available by the authors, without undue reservation, to any qualified researcher.

## Author Contributions

S-ZW worked on the cell culture, lentiviral transduction and *T. gondii* infection, siRNA assary, miRNA inhibitors and mimics transfection, qPCR, cell viability assay, and manuscript drafting. H-XW worked on the cell culture, lentiviral transduction and *T. gondii* infection, qPCR, fluorescence microscopy, bioinformatic analysis, figure drafting, manuscript drafting, and funding support. DJ was responsible for siRNA assary, miRNA inhibitors, and mimics transfection. S-ML and W-HZ worked on the cell culture and qPCR. H-JP was responsible for conception and designing, supervision of the research group, funding support, and manuscript drafting. All authors read and approved the final manuscript.

### Conflict of Interest

The authors declare that the research was conducted in the absence of any commercial or financial relationships that could be construed as a potential conflict of interest.

## Supplementary Material

Figure S1 Some human foreskin fibroblast (HFF) cells transduced with the lentiviral library showed resistance to *T. gondii* infection. About 1 × 10^7^ HFF cells treated or untreated with the lentivirus library were infected with *T. gondii* ME49 tachyzoites (at multiplicity of infection 1, MOI = 1). At 4 days post infection, more free tachyzoites were found in the lentivirus-treated group compared to the untreated group. This phenomenon may be due to the knockdown of immune-associated genes. At 10 days post infection, the cells in the untreated group were all lysed by *T. gondii*. However, there were still remaining cells in the lentivirus treated group, indicating that the specific host gene knockdown inhibited *T. gondii* infection or multiplication.

Table S1 siRNA, inhibitor, mimic, and primer sequences list.

Table S2 Information on the 36,898, 36,901, and 39,133 most abundant sgRNAs sequenced from three independent biological replicates of lentivirus treated HFF cells infected by *T. gondii* for 10 days.

Table S3 Overlapped host dependency factors screened from three independent biological replicates.

Table S4 The 2760 predicted target genes of 17 mature miRNAs.

Table S5 Detailed information on top 10 Kyoto Encyclopedia of Genes and Genomes (KEGG) enriched pathways for the 1,183 host dependency genes.

Table S6 Detailed information about seven protein–protein-interacting clusters in the protein–protein interaction (PPI) network for 1,183 host dependency genes.

## References

[B1] Agarwal V. Bell G. W. Nam J. W. Bartel D. P. (2015). Predicting effective microRNA target sites in mammalian mRNAs. eLife. 4:e05005. 10.7554/eLife.05005 26267216 PMC4532895

[B2] Baruah P. Simpson E. Dumitriu I. E. Derbyshire K. Coe D. Addey C.. (2010). Mice lacking C1q or C3 show accelerated rejection of minor H disparate skin grafts and resistance to induction of tolerance. Eur. J. Immunol. 40, 1758–1767. 10.1002/eji.200940158 20213737 PMC2988415

[B3] Black M. W. Boothroyd J. C. (2000). Lytic cycle of *Toxoplasma gondii*. Microbiol. Mol. Biol. Rev. 64, 607–623. 10.1128/mmbr.64.3.607-623.2000 10974128 PMC99006

[B4] Celis-Gutierrez J. Boyron M. Walzer T. Pandolfi P. P. Jonjic S. Olive D.. (2014). Dok1 and Dok2 proteins regulate natural killer cell development and function. EMBO J. 33, 1928–1940. 10.15252/embj.201387404 24963146 PMC4195787

[B5] Chung H. Rice C. M. (2018). T time for ADAR: ADAR1 is required for T cell self-tolerance. EMBO Rep. 19:e47237. 10.15252/embr.201847237 30467234 PMC6280789

[B6] Cook T. Roos D. Morada M. Zhu G. Keithly J. S. Feagin J. E.. (2007). Divergent polyamine metabolism in the Apicomplexa. Microbiology 153, 1123–1130. 10.1099/mic.0.2006/001768-0 17379721

[B7] Coppens I. Dunn J. D. Romano J. D. Pypaert M. Zhang H. Boothroyd J. C.. (2006). *Toxoplasma gondii* sequesters lysosomes from mammalian hosts in the vacuolar space. Cell 125, 261–274. 10.1016/j.cell.2006.01.056 16630815

[B8] Cozacov R. Halasz K. Haj T. Vadasz Z. (2017). Semaphorin 3A: is a key player in the pathogenesis of asthma. Clin. Immunol. 184, 70–72. 10.1016/j.clim.2017.05.011 28502680

[B9] Cuenca M. Sintes J. Lanyi A. Engel P. (2019). CD84 cell surface signaling molecule: an emerging biomarker and target for cancer and autoimmune disorders. Clin. Immunol. 204, 43–49. 10.1016/j.clim.2018.10.017 30522694

[B10] Dembek C. Protzer U. Roggendorf M. (2018). Overcoming immune tolerance in chronic hepatitis B by therapeutic vaccination. Curr. Opin. Virol. 30, 58–67. 10.1016/j.coviro.2018.04.003 29751272

[B11] Dong S. Corre B. Foulon E. Dufour E. Veillette A. Acuto O.. (2006). T cell receptor for antigen induces linker for activation of T cell-dependent activation of a negative signaling complex involving Dok-2, SHIP-1, and Grb-2. J. Exp. Med. 203, 2509–2518. 10.1084/jem.20060650 17043143 PMC2118126

[B12] Dweep H. Gretz N. Sticht C. (2014). miRWalk database for miRNA-target interactions. Methods Mol. Biol. 1182, 289–305. 10.1007/978-1-4939-1062-5_25 25055920

[B13] Echeverri C. J. Beachy P. A. Baum B. Boutros M. Buchholz F. Chanda S. K.. (2006). Minimizing the risk of reporting false positives in large-scale RNAi screens. Nat. Methods 3, 777–779. 10.1038/nmeth1006-777 16990807

[B14] Elliott D. A. Clark D. P. (2000). Cryptosporidium parvum induces host cell actin accumulation at the host-parasite interface. Infect. Immun. 68, 2315–2322. 10.1128/iai.68.4.2315-2322.2000 10722635 PMC97419

[B15] Fox B. A. Gigley J. P. Bzik D. J. (2004). *Toxoplasma gondii* lacks the enzymes required for de novo arginine biosynthesis and arginine starvation triggers cyst formation. Int. J. Parasitol. 34, 323–331. 10.1016/j.ijpara.2003.12.001 15003493

[B16] Gaji R. Y. Huynh M. H. Carruthers V. B. (2013). A novel high throughput invasion screen identifies host actin regulators required for efficient cell entry by *Toxoplasma gondii*. PLoS ONE 8:e64693. 10.1371/journal.pone.0064693 23741372 PMC3669402

[B17] George C. X. Ramaswami G. Li J. B. Samuel C. E. (2016). Editing of Cellular Self-RNAs by adenosine deaminase ADAR1 suppresses innate immune stress responses. J. Biol. Chem. 291, 6158–6168. 10.1074/jbc.M115.709014 26817845 PMC4813567

[B18] Gonzalez V. Combe A. David V. Malmquist N. A. Delorme V. Leroy C.. (2009). Host cell entry by apicomplexa parasites requires actin polymerization in the host cell. Cell Host Microbe 5, 259–272. 10.1016/j.chom.2009.01.011 19286135

[B19] Han Q. Lan P. Zhang J. Zhang C. Tian Z. (2013). Reversal of hepatitis B virus-induced systemic immune tolerance by intrinsic innate immune stimulation. J. Gastroenterol. Hepatol. 28(Suppl. 1), 132–137. 10.1111/jgh.12034 23855309

[B20] Hong Y. Manoharan I. Suryawanshi A. Shanmugam A. Swafford D. Ahmad S.. (2016). Deletion of LRP5 and LRP6 in dendritic cells enhances antitumor immunity. Oncoimmunol. 5:e1115941. 10.1080/2162402X.2015.1115941 27141399 PMC4839371

[B21] Horst A. K. Najjar S. M. Wagener C. Tiegs G. (2018). CEACAM1 in liver injury, metabolic and immune regulation. Int. J. Mol. Sci. 19:E3110. 10.3390/ijms19103110 30314283 PMC6213298

[B22] Huang Y. H. Zhu C. Kondo Y. Anderson A. C. Gandhi A. Russell A.. (2015). CEACAM1 regulates TIM-3-mediated tolerance and exhaustion. Nature 517, 386–390. 10.1038/nature13848 25363763 PMC4297519

[B23] Kalim K. W. Yang J. Q. Li Y. Meng Y. Zheng Y. Guo F. (2018). Reciprocal regulation of glycolysis-driven Th17 pathogenicity and regulatory T cell stability by Cdc42. J. Immunol. 200, 2313–2326. 10.4049/jimmunol.1601765 29440353 PMC5860966

[B24] Kasler H. G. Lim H. W. Mottet D. Collins A. M. Lee I. S. Verdin E. (2012). Nuclear export of histone deacetylase 7 during thymic selection is required for immune self-tolerance. EMBO J. 31, 4453–4465. 10.1038/emboj.2012.295 23103766 PMC3512390

[B25] Kieffer F. Wallon M. (2013). Congenital toxoplasmosis. Handb. Clin. Neurol. 112, 1099–1101. 10.1016/B978-0-444-52910-7.00028-3 23622316

[B26] Kim H. S. Sohn H. Jang S. W. Lee G. R. (2019). The transcription factor NFIL3 controls regulatory T-cell function and stability. Exp. Mol. Med. 51:80. 10.1038/s12276-019-0280-9 31311918 PMC6802641

[B27] Kuleshov M. V. Jones M. R. Rouillard A. D. Fernandez N. F. Duan Q. Wang Z.. (2016). Enrichr: a comprehensive gene set enrichment analysis web server 2016 update. Nucleic Acids Res. 44, W90–W97. 10.1093/nar/gkw377 27141961 PMC4987924

[B28] Larrubia J. R. Lokhande M. U. Garcia-Garzon S. Miquel J. Subira D. Sanz-de-Villalobos E. (2013). Role of T cell death in maintaining immune tolerance during persistent viral hepatitis. World J. Gastroenterol. 19, 1877–1889. 10.3748/wjg.v19.i12.1877 23569333 PMC3613103

[B29] Li X. L. Wei H. X. Zhang H. Peng H. J. Lindsay D. S. (2014). A meta analysis on risks of adverse pregnancy outcomes in *Toxoplasma gondii* infection. PLoS ONE 9:e97775. 10.1371/journal.pone.0097775 24830795 PMC4022675

[B30] Liu Q. Zhou H. Langdon W. Y. Zhang J. (2014). E3 ubiquitin ligase Cbl-b in innate and adaptive immunity. Cell Cycle 13, 1875–1884. 10.4161/cc.29213 24875217 PMC4111751

[B31] Liu Y. C. Penninger J. Karin M. (2005). Immunity by ubiquitylation: a reversible process of modification. Nat. Rev. Immunol. 5, 941–952. 10.1038/nri1731 16322747 PMC7096784

[B32] Lozano E. Dominguez-Villar M. Kuchroo V. Hafler D. A. (2012). The TIGIT/CD226 axis regulates human T cell function. J. Immunol. 188, 3869–3875. 10.4049/jimmunol.1103627 22427644 PMC3324669

[B33] Ma H. Dang Y. Wu Y. Jia G. Anaya E. Zhang J.. (2015). A CRISPR-based screen identifies genes essential for West-Nile-virus-induced cell death. Cell Rep. 12, 673–683. 10.1016/j.celrep.2015.06.049 26190106 PMC4559080

[B34] Marquet J. Lasoudris F. Cousin C. Puiffe M. L. Martin-Garcia N. Baud V.. (2010). Dichotomy between factors inducing the immunosuppressive enzyme IL-4-induced gene 1 (IL4I1) in B lymphocytes and mononuclear phagocytes. Eur. J. Immunol. 40, 2557–2568. 10.1002/eji.201040428 20683900 PMC3001484

[B35] Menendez M. T. Teygong C. Wade K. Florimond C. Blader I. J. (2015). siRNA screening identifies the host Hexokinase 2 (HK2) gene as an important hypoxia-inducible transcription factor 1 (HIF-1) target gene in *Toxoplasma gondii*-infected cells. mBio 6:e00462. 10.1128/mBio.00462-15 26106078 PMC4479703

[B36] Miyakawa K. Matsunaga S. Yokoyama M. Nomaguchi M. Kimura Y. Nishi M.. (2019). PIM kinases facilitate lentiviral evasion from SAMHD1 restriction via Vpx phosphorylation. Nat. Commun. 10:1844. 10.1038/s41467-019-09867-7 31015445 PMC6479052

[B37] Mori T. Suzuki-Yamazaki N. Takaki S. (2018). Lnk/Sh2b3 regulates adipose inflammation and glucose tolerance through group 1 ILCs. Cell Rep. 24, 1830–1841. 10.1016/j.celrep.2018.07.036 30110639

[B38] Moser L. A. Pollard A. M. Knoll L. J. (2013). A genome-wide siRNA screen to identify host factors necessary for growth of the parasite *Toxoplasma gondii*. PLoS ONE 8:e68129. 10.1371/journal.pone.0068129 23840822 PMC3695992

[B39] Muniz-Feliciano L. Van Grol J. Portillo J. A. Liew L. Liu B. Carlin C. R.. (2013). *Toxoplasma gondii*-induced activation of EGFR prevents autophagy protein-mediated killing of the parasite. PLoS Pathog. 9:e1003809. 10.1371/journal.ppat.1003809 24367261 PMC3868508

[B40] Munter S. Way M. Frischknecht F. (2006). Signaling during pathogen infection. Sci. STKE 2006:re5. 10.1126/stke.3352006re5 16705131

[B41] Na R. H. Zhu G. H. Luo J. X. Meng X. J. Cui L. Peng H. J.. (2013). Enzymatically active Rho and Rac small-GTPases are involved in the establishment of the vacuolar membrane after *Toxoplasma gondii* invasion of host cells. BMC Microbiol. 13:125. 10.1186/1471-2180-13-125 23721065 PMC3681593

[B42] O'Connell M. A. Mannion N. M. Keegan L. P. (2015). The Epitranscriptome and Innate Immunity. PLoS Genet. 11:e1005687. 10.1371/journal.pgen.1005687 26658668 PMC4675516

[B43] O'Neill L. A. (2008). When signaling pathways collide: positive and negative regulation of toll-like receptor signal transduction. Immunity 29, 12–20. 10.1016/j.immuni.2008.06.004 18631453

[B44] Overacre-Delgoffe A. E. Chikina M. Dadey R. E. Yano H. Brunazzi E. A. Shayan G.. (2017). Interferon-γ drives treg fragility to promote anti-tumor immunity. Cell 169, 1130–1141.e11. 10.1016/j.cell.2017.05.005 28552348 PMC5509332

[B45] Paraskevopoulou M. D. Georgakilas G. Kostoulas N. Vlachos I. S. Vergoulis T. Reczko M.. (2013). DIANA-microT web server v5.0: service integration into miRNA functional analysis workflows. Nucleic Acids Res. 41, W169–W173. 10.1093/nar/gkt393 23680784 PMC3692048

[B46] Park R. J. Wang T. Koundakjian D. Hultquist J. F. Lamothe-Molina P. Monel B.. (2017). A genome-wide CRISPR screen identifies a restricted set of HIV host dependency factors. Nat. Genet. 49, 193–203. 10.1038/ng.3741 27992415 PMC5511375

[B47] Park Y. (2007). Functional evaluation of the type 1 diabetes (T1D) susceptibility candidate genes. Diabetes Res. Clin. Pract. 77(Suppl. 1), S110–S115. 10.1016/j.diabres.2007.01.043 17448564

[B48] Patel J. C. Galan J. E. (2005). Manipulation of the host actin cytoskeleton by Salmonella–all in the name of entry. Curr. Opin. Microbiol. 8, 10–15. 10.1016/j.mib.2004.09.001 15694851

[B49] Peng H. J. Chen X. G. Lindsay D. S. (2011). A review: competence, compromise, and concomitance-reaction of the host cell to *Toxoplasma gondii* infection and development. J. Parasitol. 97, 620–628. 10.1645/GE-2712.1 21506833

[B50] Qin K. Han C. Zhang H. Li T. Li N. Cao X. (2017). NAD(+) dependent deacetylase Sirtuin 5 rescues the innate inflammatory response of endotoxin tolerant macrophages by promoting acetylation of p65. J. Autoimmun. 81, 120–129. 10.1016/j.jaut.2017.04.006 28461090

[B51] Sanjana N. E. Shalem O. Zhang F. (2014). Improved vectors and genome-wide libraries for CRISPR screening. Nat. Methods 11, 783–784. 10.1038/nmeth.3047 25075903 PMC4486245

[B52] Sarkar S. Bristow C. A. Dey P. Rai K. Perets R. Ramirez-Cardenas A.. (2017). PRKCI promotes immune suppression in ovarian cancer. Genes Dev. 31, 1109–1121. 10.1101/gad.296640.117 28698296 PMC5538434

[B53] Scarlata C. M. Celse C. Pignon P. Ayyoub M. Valmori D. (2015). Differential expression of the immunosuppressive enzyme IL4I1 in human induced Aiolos+, but not natural Helios+, FOXP3+ Treg cells. Eur. J. Immunol. 45, 474–479. 10.1002/eji.201444897 25446972

[B54] Schwartzman J. D. Pfefferkorn E. R. (1982). *Toxoplasma gondii*: purine synthesis and salvage in mutant host cells and parasites. Exp. Parasitol. 53, 77–86. 10.1016/0014-4894(82)90094-7 7198995

[B55] Shalem O. Sanjana N. E. Hartenian E. Shi X. Scott D. A. Mikkelson T.. (2014). Genome-scale CRISPR-Cas9 knockout screening in human cells. Science 343, 84–87. 10.1126/science.1247005 24336571 PMC4089965

[B56] Shi J. Wang E. Milazzo J. P. Wang Z. Kinney J. B. Vakoc C. R. (2015). Discovery of cancer drug targets by CRISPR-Cas9 screening of protein domains. Nat. Biotechnol. 33, 661–667. 10.1038/nbt.3235 25961408 PMC4529991

[B57] Snell L. M. McGaha T. L. Brooks D. G. (2017). Type I interferon in chronic virus infection and cancer. Trends Immunol. 38, 542–557. 10.1016/j.it.2017.05.005 28579323 PMC8059441

[B58] Steinhart Z. Pavlovic Z. Chandrashekhar M. Hart T. Wang X. Zhang X. (2017). Genome-wide CRISPR screens reveal a Wnt-FZD5 signaling circuit as a druggable vulnerability of RNF43-mutant pancreatic tumors. Nat. Med. 23, 60–68. 10.1038/nm.4219 27869803

[B59] Sticht C. De La Torre C. Parveen A. Gretz N. (2018). miRWalk: An online resource for prediction of microRNA binding sites. PLoS ONE 13:e0206239. 10.1371/journal.pone.0206239 30335862 PMC6193719

[B60] Tang R. Langdon W. Y. Zhang J. (2019). Regulation of immune responses by E3 ubiquitin ligase Cbl-b. Cell. Immunol. 340:103878. 10.1016/j.cellimm.2018.11.002 30442330 PMC6504630

[B61] Teymouri M. Pirro M. Fallarino F. Gargaro M. Sahebkar A. (2018). IL-35, a hallmark of immune-regulation in cancer progression, chronic infections and inflammatory diseases. Int. J. Cancer 143, 2105–2115. 10.1002/ijc.31382 29574719

[B62] Tsubata T. (2017). B-cell tolerance and autoimmunity. F1000Res 6:391. 10.12688/f1000research.10583.1 28408984 PMC5373417

[B63] Vadasz Z. Toubi E. (2014). Semaphorins: their dual role in regulating immune-mediated diseases. Clin. Rev. Allergy Immunol. 47, 17–25. 10.1007/s12016-013-8360-4 23397481

[B64] Verdin E. Dequiedt F. Kasler H. (2004). HDAC7 regulates apoptosis in developing thymocytes. Novartis Found Symp. 259, 115–29; discussion 129–31,163–9. 10.1002/0470862637.ch8 15171250

[B65] Wei H. Zhou L. Wu S. Li D. Deng S. Peng H. (2019). Host cell Rac1 GTPase facilitates *Toxoplasma gondii* invasion. Sci. China Life Sci. 10.1007/s11427-019-9564-0. [Epub ahead of print]. 31222548

[B66] Wong E. B. Soni C. Chan A. Y. Domeier P. P. Shwetank Abraham T.. (2015). B cell-intrinsic CD84 and Ly108 maintain germinal center B cell tolerance. J. Immunol. 194, 4130–4143. 10.4049/jimmunol.1403023 25801429 PMC4402266

[B67] Yamada A. Arakaki R. Saito M. Kudo Y. Ishimaru N. (2017). Dual role of Fas/FasL-mediated signal in peripheral immune tolerance. Front. Immunol. 8:403. 10.3389/fimmu.2017.00403 28424702 PMC5380675

[B68] Yau E. H. Kummetha I. R. Lichinchi G. Tang R. Zhang Y. Rana T. M. (2017). Genome-wide CRISPR screen for essential cell growth mediators in mutant KRAS colorectal cancers. Cancer Res. 77, 6330–6339. 10.1158/0008-5472.CAN-17-2043 28954733 PMC5690866

[B69] Yoshimura A. Muto G. (2011). TGF-β function in immune suppression. Curr. Top. Microbiol. Immunol. 350, 127–147. 10.1007/82_2010_87 20680806

[B70] Yu M. Chen Y. He Y. Podd A. Fu G. Wright J. A.. (2012). Critical role of B cell lymphoma 10 in BAFF-regulated NF-kappaB activation and survival of anergic B cells. J. Immunol. 189, 5185–5193. 10.4049/jimmunol.1102952 23087406 PMC3786778

[B71] Yu X. Harden K. Gonzalez L. C. Francesco M. Chiang E. Irving B.. (2009). The surface protein TIGIT suppresses T cell activation by promoting the generation of mature immunoregulatory dendritic cells. Nat. Immunol. 10, 48–57. 10.1038/ni.1674 19011627

[B72] Zhang R. Miner J. J. Gorman M. J. Rausch K. Ramage H. White J. P.. (2016). A CRISPR screen defines a signal peptide processing pathway required by flaviviruses. Nature 535, 164–168. 10.1038/nature18625 27383988 PMC4945490

[B73] Zhu C. Sakuishi K. Xiao S. Sun Z. Zaghouani S. Gu G. (2015). An IL-27/NFIL3 signalling axis drives Tim-3 and IL-10 expression and T-cell dysfunction. Nat. Commun. 6:6072 10.1038/ncomms7072 25614966 PMC4311884

