# Supplementary figures and images for: Genome-Wide CRISPR Screen Identifies Host Factors Required by Toxoplasma gondii Infection
Source: Front Cell Infect Microbiol. 2020 Jan 22;9:460. doi: 10.3389/fcimb.2019.00460 (PMC6987080; doi:10.3389/fcimb.2019.00460)

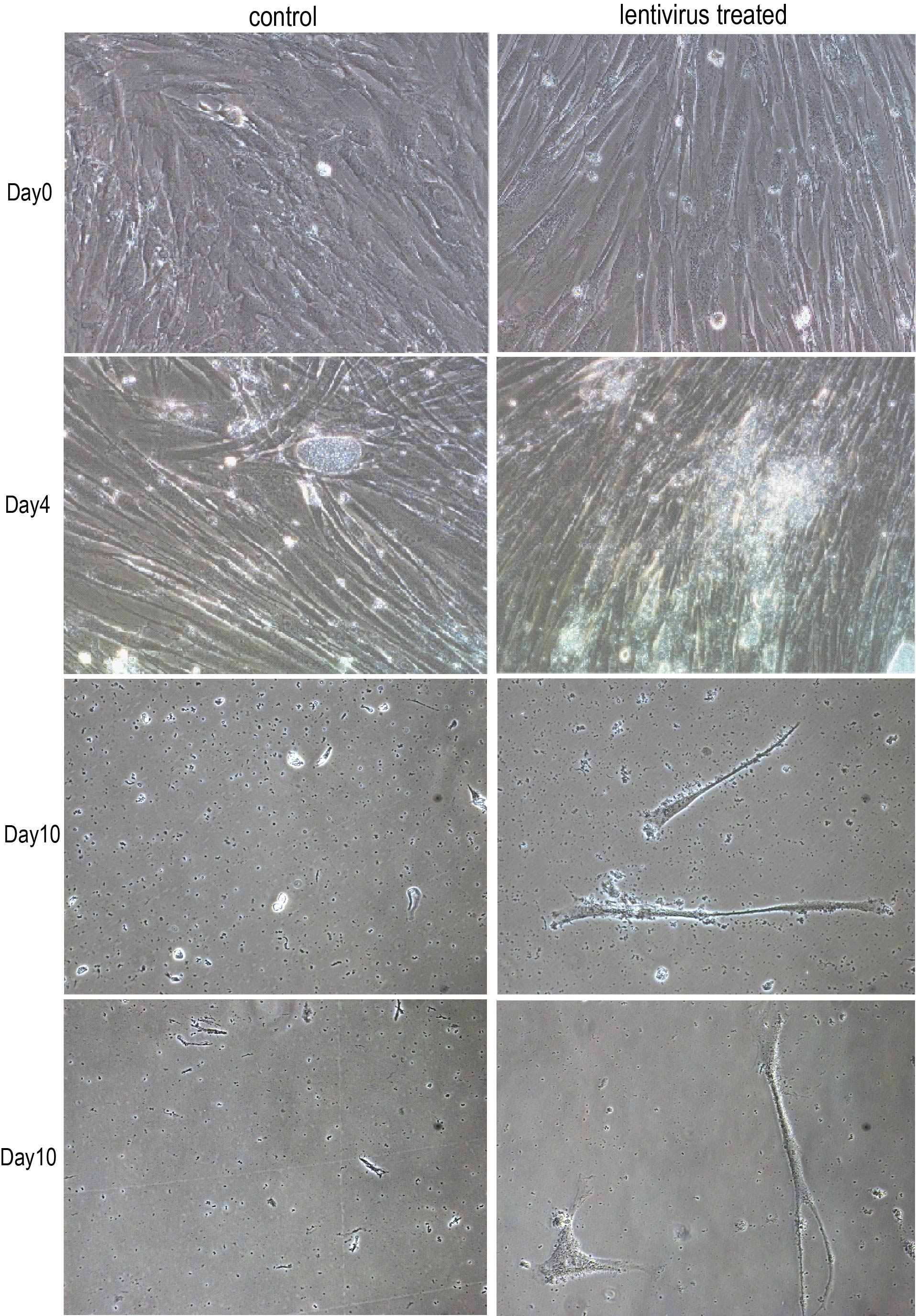

Supplement: Figure S1 — Some human foreskin fibroblast (HFF) cells transduced with the lentiviral library showed resistance to T. gondii infection. About 1 × 10^7^ HFF cells treated or untreated with the lentivirus library were infected with T. gondii ME49 tachyzoites (at multiplicity of infection 1, MOI = 1). At 4 days post infection, more free tachyzoites were found in the lentivirus-treated group compared to the untreated group. This phenomenon may be due to the knockdown of immune-associated genes. At 10 days post infection, the cells in the untreated group were all lysed by T. gondii. However, there were still remaining cells in the lentivirus treated group, indicating that the specific host gene knockdown inhibited T. gondii infection or multiplication. [file Image_1.JPEG]
