# Supplementary material for: Genome-Wide CRISPR Screen Identifies Host Factors Required by Toxoplasma gondii Infection
Source: Front Cell Infect Microbiol. 2020 Jan 22;9:460. doi: 10.3389/fcimb.2019.00460 (PMC6987080; doi:10.3389/fcimb.2019.00460)
Supplement: Table S1 — siRNA, inhibitor, mimic, and primer sequences list. [file Table_1.DOCX]

Table S1 siRNA, inhibitor, mimic and primer sequences list

| **Name** | **sequences** |
| --- | --- |
| USP17L24 siRNA1 | 5’-CACTCTACTTGAGAGGTGA-3’ |
| USP17L24 siRNA2 | 5’-CTCTACTTGAGAGGTGAGT-3’ |
| USP17L24 siRNA3 | 5’-GACACCGACAGGCGAGCAA-3’ |
| USP17L24 qPCR primer | F:5’-ACCTCTCGTCTATGTCCTCTATG-3’; R:5’-GACCTCGGCATCATCCATTT-3’ |
| USP19 siRNA1 | 5’-GCACAGTGACAACGGTAGA-3’ |
| USP19 siRNA2 | 5’-ATACGCTCCTCTGCTTTGA-3’ |
| USP19 siRNA3 | 5’-CAGGGACACCTCAAGAGTA-3’ |
| USP19 qPCR primer | F:5’-TAAATCCAAGGCACGATCTGAGG-3’; R:5’-GCTTTGGGGTTACATGCTCCA-3’ |
| HDAC7 siRNA1 | 5’-CTCACGTCCAGGTGATCAA-3’ |
| HDAC7 siRNA2 | 5’-CTGCGCTATAAGCCCAAGA-3’ |
| HDAC7 siRNA3 | 5’-AGATACCCTCGGCTGAAGA-3’ |
| HDAC7 qPCR primer | F:5’-CAGGACCCATCCGACTCTG-3’; R:5’-GGACTGGGCAAAGTGGAAGG-3’ |
| ULK1 siRNA1 | 5’-GCCTGTTCTACGAGAAGAA-3’ |
| ULK1 siRNA2 | 5’-CGCCTGTTCTACGAGAAGA-3’ |
| ULK1 siRNA3 | 5’-ACCAGCGCATTGAGCGAAA-3’ |
| ULK1 qPCR primer | F:5’-GGCAAGTTCGAGTTCTCCCG-3’; R:5’-CGACCTCCAAATCGTGCTTCT-3’ |
| PIM1 siRNA1 | 5’-GCCCTGAGACCATCAGATA-3’ |
| PIM1 siRNA2 | 5’-GGATCCTGCTGTATGATAT-3’ |
| PIM1 siRNA3 | 5’-GTGGAGATATTCCTTTCGA-3’ |
| PIM1 qPCR primer | F:5’-GAGAGGCCCGACAGTTTCG-3’; R:5’-CTCCCCTTTCCGATGAAGT-3’ |
| ENPP5 siRNA1 | 5’-GTCATACCCTTATTTCATA-3’ |
| ENPP5 siRNA2 | 5’-GCAGCATTATAGTGATTGT-3’ |
| ENPP5 siRNA3 | 5’-CCAGAAAGGTGGCATTACA-3’ |
| ENPP5 qPCR primer | F:5’-AGTTTTGGGAAGAAGCGACAC-3’; R:5’-GGCATGTAATGAGTAGGAAAGCG-3’ |
| CBLB siRNA1 | 5’-GGAGATAACTTTCGTATCA-3’ |
| CBLB siRNA2 | 5’-GGAGCATCCTCCGAGAATT-3’ |
| CBLB siRNA3 | 5’-GGACGACGATGATGATCGT-3’ |
| CBLB qPCR primer | F:5’-CGGCAATATCCTACAGACCATAC-3’; R:5’-CCGCCCATCAGGATAAAGATAA-3’ |
| GAPDH qPCR primer | F:5’-GGAGCGAGATCCCTCCAAAAT-3’; R:5’-GGCTGTTGTCATACTTCTCATGG-3’ |
| Actin qPCR primer | F:5’-GGACCTGACTGACTACCTCAT-3’; R:5’-CGTAGCACAGCTTCTCCTTAAT-3’ |
| miR-1270 inhibitor | 5’-ACACAGCUCUUCCAUAUCUCCAG-3’ |
| miR-22-5p inhibitor | 5’-UAAAGCUUGCCACUGAAGAACU-3’ |
| miR-3065-5p inhibitor | 5’-UCCAGCAUCAGUGAUUUUGUUGA-3’ |
| miR-642a-5p inhibitor | 5’-CAAGACACAUUUGGAGAGGGAC-3’ |
| miR-656-5p inhibitor | 5’-UGAACACCUCACAGGCAACCU-3’ |
| inhibitor NC-FAM | 5’-CAGUACUUUUGUGUAGUACAA-3 |
| inhibitor NC | 5’-CAGUACUUUUGUGUAGUACAA-3 |
| miR-1270 mimics | 5’-CUGGAGAUAUGGAAGAGCUGUGU-3’; 5’-ACAGCUCUUCCAUAUCUCCAGUU-3’ |
| miR-22-5p mimics | 5’-AGUUCUUCAGUGGCAAGCUUUA-3’; 5’-AAGCUUGCCACUGAAGAACUUU-3’ |
| miR-3065-5p mimics | 5’-UCAACAAAAUCACUGAUGCUGGA-3’; 5’-CAGCAUCAGUGAUUUUGUUGAUU-3’ |
| miR-642a-5p mimics | 5’-GUCCCUCUCCAAAUGUGUCUUG-3’; 5’-AGACACAUUUGGAGAGGGACUU-3’ |
| miR-656-5p mimics | 5’-AGGUUGCCUGUGAGGUGUUCA-3’; 5’-AACACCUCACAGGCAACCUUU-3’ |
| Mimic NC-FAM | 5’-UUCUCCGAACGUGUCACGUTT-3’; 5’-ACGUGACACGUUCGGAGAATT-3’ |
| Mimic NC | 5’-UUCUCCGAACGUGUCACGUTT-3’; 5’-ACGUGACACGUUCGGAGAATT-3’ |
