# Supplementary material for: Genome-Wide CRISPR Screen Identifies Host Factors Required by Toxoplasma gondii Infection
Source: Front Cell Infect Microbiol. 2020 Jan 22;9:460. doi: 10.3389/fcimb.2019.00460 (PMC6987080; doi:10.3389/fcimb.2019.00460)
Supplement: Table S5 — Detailed information on top 10 Kyoto Encyclopedia of Genes and Genomes (KEGG) enriched pathways for the 1,183 host dependency genes. [file Table_5.DOCX]

**Table S5. The detailed information for the top 10 enriched pathways.**

| **Term** | **number** | **P-value** | **Genes** |
| --- | --- | --- | --- |
| PI3K-Akt signaling pathway | 30 | 0.030633 | IFNA5;PHLPP2;CDKN1A;PHLPP1;FLT3;ITGB4;IFNA2;PDGFA;PIK3CB;BRCA1;IL2RG;EGFR;THBS4;INS;CREB3L4;FGF20;RPS6;PPP2R3A;PGF;EFNA1;PIK3CA;IL2RA;GNB2;COL6A2;BCL2;ITGA7;COL6A3;SGK2;SOS2;FGF10 |
| Regulation of actin cytoskeleton | 23 | 0.004136 | ITGB4;ARPC1A;PDGFA;IQGAP1;PIK3CB;ITGAE;SSH3;EGFR;MYL12A;VAV2;MYL12B;INS;CDC42;PIK3CA;ITGAD;ITGAX;FGF20;ITGA7;ARHGEF1;WASF1;PPP1R12C;SOS2;FGF10 |
| Human cytomegalovirus infection | 22 | 0.014355 | IFNA5;CDKN1A;IFNA2;ADCY2;PIK3CB;TNF;EGFR;ADCY5;MAPK11;ADCY9;PIK3CA;IRF3;CREB3L4;GNB2;PRKACG;CCL3;CTNNB1;FAS;ARHGEF1;E2F3;PRKACA;SOS2 |
| Rap1 signaling pathway | 21 | 0.010664 | PRKCI;FPR1;PDGFA;ADCY2;PIK3CB;SIPA1L3;EGFR;PGF;VAV2;ADCY5;INS;EFNA1;CDC42;MAPK11;SIPA1L1;ADCY9;PIK3CA;FGF20;CTNNB1;PRKD2;FGF10 |
| Focal adhesion | 18 | 0.048286 | ITGB4;PDGFA;PIK3CB;EGFR;PGF;THBS4;MYL12A;VAV2;MYL12B;CDC42;PIK3CA;COL6A2;BCL2;CTNNB1;ITGA7;COL6A3;PPP1R12C;SOS2 |
| Relaxin signaling pathway | 16 | 0.004265 | SMAD3;ADCY2;PIK3CB;EGFR;ADCY5;MAPK11;EDNRB;ADCY9;PIK3CA;CREB3L4;GNB2;PRKACG;NOS1;PRKACA;SOS2;INSL5 |
| mTOR signaling pathway | 16 | 0.018068 | FZD1;PRR5;WNT7B;RPS6;PIK3CB;TNF;LRP6;INS;RPS6KA6;WNT11;PIK3CA;RRAGB;ATP6V1B2;ULK1;ATP6V1G3;SOS2 |
| Hepatitis C | 16 | 0.021332 | IFNA5;CDKN1A;IFNA2;PIK3CB;TNF;EGFR;PIAS1;CLDN6;PIK3CA;IRF3;CLDN7;CLDN19;FAS;CTNNB1;E2F3;SOS2 |
| JAK-STAT signaling pathway | 16 | 0.030672 | IFNA5;CDKN1A;IFNA2;IL13;PDGFA;IL20RB;PIK3CB;IL2RG;EGFR;PIAS1;PIK3CA;IL2RA;BCL2;PIM1;SOCS6;SOS2 |
| Hepatitis B | 16 | 0.032219 | IFNA5;CDKN1A;SMAD3;MAP3K1;IFNA2;PIK3CB;TNF;MAPK11;PIK3CA;IRF3;CREB3L4;BCL2;FAS;TAB2;E2F3;SOS2 |
